# Peripheral Venous Pressure as a Predictor of Central Venous Pressure in Continuous Monitoring in Children

**Published:** 2011-05-01

**Authors:** H Amoozgar, Gh H Ajami, M Borzuoee, A A Amirghofran, P Ebrahimi

**Affiliations:** 1 Department of Pediatrics, Division of Pediatric Cardiology, Shiraz University of Medical Sciences, Shiraz, Iran; 2 Department of Cardiac Surgery, Shiraz University of Medical Sciences, Shiraz, Iran

**Keywords:** Peripheral venous pressure, Central venous pressure, Monitoring, Children

## Abstract

**Background:**

Measurement of central venous pressure (CVP) is a reliable method for evaluating intravascular volume status and cardiac function; however it is an invasive and expensive method that may result in some complications such as arterial puncture, pneumothorax and development of infections. This study was performedto compare CVP measurements between central and peripheral catheters in infant and children with congenital heart disease.

**Methods:**

The CVP and peripheral venous pressure (PVP) were measured simultaneously in 30 patients within 10 consecutive hours.

**Results:**

The mean difference between CVP and PVP was 1.48±0.98 mmHg. The linear regression equation showed that CVP was 0.374+0.774 PVP (r(2) = 0.725).

**Conclusion:**

PVP measured from a peripheral intravenous catheter in infants and children with congenital heart disease is an accurate estimation of CVP and its changes has good concordance with CVP over a long period of time.

## Introduction

Pediatric patients, like older patients undergoing major cardiac surgery, frequently require monitoring of central venous pressure (CVP) to obtain information about intravascular volume status and cardiac function.[1] Whether CVP monitoring improves the patient outcome has not been proved, however there are some risks including arterial puncture, pneumothorax , infection, etc. associated with monitoring that often outweighs the benefits to the patients.[1][2] There are also some patients among them surgical sites or altered anatomy due to previous surgery or radiation prohibits CVP catheter placement.[1][3][4][5] Under the latter conditions, although inserting the catheter into jugular or subclavian veins is not impossible but is associated with significant risks.

Based on these restrictions, many studies were carried out to show the reliability and consistence correlation between CVP and peripheral venous pressure (PVP) measurements.[1][3][4] It implies that in emergency conditions, the estimation of CVP is possible via measurement of peripheral intravenous catheter. Previous studies have not evaluated the concordance between these methods during a long period of time. The goal of the present study was to determine whether a reliable association exists between changes in CVP and PVP in varied hemodynamic status (eg; dehydration, bleeding or volume overload) in pediatric cardiac surgery patients during the first 10 hours after cardiac surgery.

## Material and Methods

The study included 30 pediatric patients (age ranges=10 days to 18 years), with different congenital heart disease hospitals affiliated to Shiraz University of Medical Science in Shiraz, Fars Province, southern Iran. The study complies with the Declaration of Helsinki, and approved by the Institutional Review Board of Ethics at the Shiraz University of Medical Sciences and a written informed consent was obtained from all guardians.

CVP access was obtained using a 6 or 8 French Double-lumen, Arrow International catheter with placement via the left or right internal jugular or subclavian vein. Tip of central venous catheter was inserted at the junction of the superior vena cava and right atrium in chest x-ray. The peripheral measurement of CVP was obtained from a peripheral intravenous (IV) site using a standard IV catheter (18-20-22 gauges). Central venous pressure was measured from both the central venous catheter and the peripheral IV catheters using Mindray PM 9000 monitors equipped with Medex (Kensington, MD, USA) invasive blood pressure monitoring transducers, which were zeroed at the phlebostatic axis. Continuity of the PVP catheter with the downstream venous system was demonstrated at the beginning of each measurement by observin coincident pressure changes in the PVP waveform during circumferential, proximal arm occlusion. Simultaneous measurements of CVP from central and peripheral venous catheters were made hourly for 10 consecutive hours after cardiac surgery. Age, weight, height, site of VP and PVP and IV catheter size from each patient were recorded. The ifferences between the central and peripheral CVP were evaluated using paired t test. The predictability of CVP by PVP was examined using linear regression analysis at a p value of ≤ 0.05. The analysis was erformed using SPSS software (version 15, Chicago, IL, USA).

## Results

Among the 30 patients in this study, the age range of participants (20 males and 10 females) was from 10 days to 18 years and their weight ranged from 2.6 to 55 kg. The patients had the following diagnosis: Tetralogy of fallot 14, pulmonary atresia and ventricular septal defect 4, atrial septal defect and pulmonary stenosis 4, patent ductus arteriosus and pulmonary hypertension 7 and severe aortic insufficiency 1.

**Fig. 1 :**
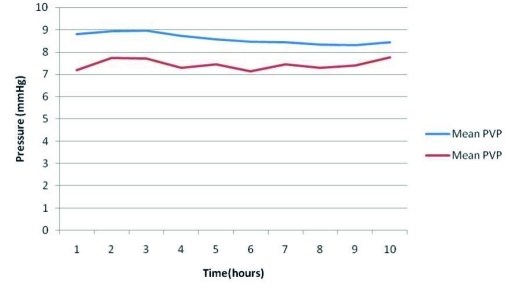
Linear regression plot of PVP versus CVP during 10 hours with 95% confidence interval

The predictability of CVP by PVP was tested by applying the linear regression which is shown in Figure 1. This regression formula reveals a reliable and significant association between CVP and PVP [CVP=0.374+0.774 PVP (r(2)=0.725, p=0.001)]. The overall mean difference between CVP and PVP was 1.48±0.98 mmHg.

**Fig. 2 :**
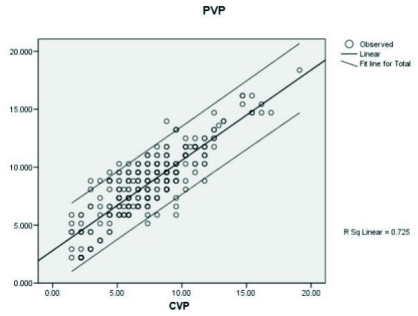
The top tracing shows the mean PVP and the bottom tracing is the simultaneous mean CVP. The distance between the two tracings shows the difference of pressure over a long period of time which remains almost constant.

**Fig. 3 :**
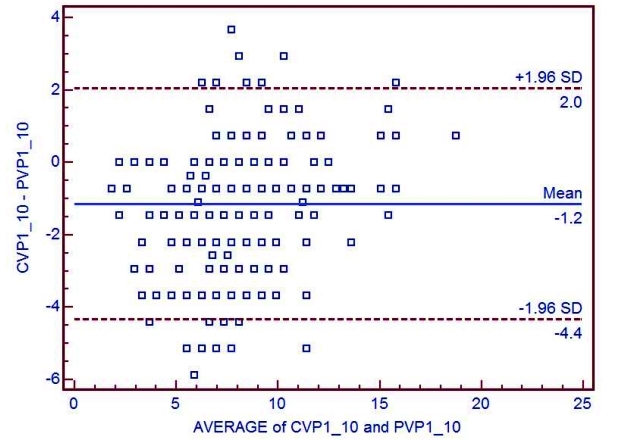
Bland-Altman diagrams for the comparison between CVP and PVP measurements during 10 hours in 30 patients. The dotted horizontal line indicates perfect agreement (difference of -1.2), the dotted lines indicate a clinically relevant difference of plus or minus 1.96. standard deviation (SD).

The mean difference between CVP and PVP in each hour was shown in Figure 2. For estimation of agreement between CVP and PVP during the 10 hours period, Bland-Altman diagram was used. This diagram showed a perfect agreement (difference of -1.2, with standard deviation of 1.96) (Figure 3).

## Discussion

Previous studies comparing CVP measured from central and peripheral access in adult patients have shown a consistent correlation between CVP and PVP measurement. However studies in pediatric groups are limited and show controversial results.[1][6][7][8]

Knowledge of the relationship between the central and peripheral measurements of CVP dates back to middle of the twentieth century, when a gradient of 4 to 7 mmHg was demonstrated from the vein of the upper extremity to the right atrium.[9] Amar et al. demonstrated a consistent correlation between CVP measured from a peripheral IV catheter and the one measured from a central line intra-operatively during both mechanical ventilation and spontaneous ventilation postoperatively.[1] Similar results were reported by Munis et al. in intra-operative cohort adult patients during neurosurgical procedures.[7]

Tabias and Johnson demonstrated that CVP could be estimated from a peripheral IV site in most infants and children.[8] Clunie et al. showed that PVP could predict CVP poorly in pediatric patients while our previous study showed statistically significant correlation between CVP and PVP in pediatric patients with congenital heart diseases.[6][10] Previous studies have shown a good correlation between isolated PVP and CVP but continuous monitoring of CVP during a long period of time and its changes is more clinically important. The present study was designed to show their changes.

According to the present results, we can estimate CVP through simultaneous measurement of PVP in specific conditions. Since the difference between CVP and PVP measurements remain almost in constant range over a period of time, the estimation of changes occurring in CVP via changes in PVP is possible. Therefore, evaluation of hemodynamic changes occurring with dehydration or volume overload can be made by measuring PVP.

In this study, the site of peripheral IV catheter in 3 patients was in lower extremities and in the rest in the upper extremities. The placement site of central venous access was left or right internal jugular or subclavian vein. Neither the site of peripheral catheter placement in the arm nor the site of central venous access could affect the result.

The measurement of PVP is a non-invasive and cost-effective procedure for pediatric patients and can predict CVP when instruments and conditions are impractical for direct measurement of CVP. The PVP and CVP changes remain in concordance overtime due to continuous monitoring of PVP used for proper estimation of CVP.
